# THE EFFECT OF MENTAL HEALTH ON DETROIT RESIDENCE IN OLDER ADULTS DURING THE COVID-19 PANDEMIC

**DOI:** 10.1093/geroni/igad104.0616

**Published:** 2023-12-21

**Authors:** Ivana Vaughn, Sean Yaphe, Lakshmi Sundaresan, Jonathan Freedman, Samuel Jesse Weinberg, Lois Lamerato, Katarzyna Budzynska

**Affiliations:** Henry Ford Health, Detroit, Michigan, United States; Clinique L’Actuel, Montreal, Quebec, Canada; Henry Ford Health, Detroit, Michigan, United States; Henry Ford Health, Detroit, Michigan, United States; Don Mills Family Health Team, Toronto, Ontario, Canada; Henry Ford Health, Detroit, Michigan, United States; Henry Ford Health, Detroit, Michigan, United States

## Abstract

The COVID-19 pandemic had a tremendous impact on global experience of anxiety and depression. Due to social isolation and quarantine, older adults faced unique challenges that disproportionately increase their risk for health outcomes. The purpose of this retrospective study was to examine the relationship between Detroit versus non-Detroit residence and incident mental health disorders. The patient population included older adults (age 65 and older) from a large metropolitan urban health system with patient encounters between December 2018 - June 2021. The primary predictor, Detroit metropolitan status, was based on zip code. Outcome variables, incident depression, anxiety, and composite mental health disorders, were based on screening measures (PHQ-2, PHQ-9 and GAD-7 scores), ICD diagnosis, medical prescriptions, and behavioral health referrals. Fully adjusted logistic regression analysis derived odds ratios of incident outcomes in Detroit versus non-Detroit patients. A total of 5,300 older adult patients were included in this study. Older adults residing in Detroit had significantly reduced odds of incident anxiety compared to non-Detroit residents (OR:0.45; 95%CI:0.26,0.75, p=0.003). Incident depression and composite mental health outcomes also showed protective effects in Detroit patients; however, these outcomes were not statistically significant. Overall, patients residing in Detroit had 55% decreased odds of developing anxiety during the pandemic. Additional research should explore facilitators and barriers to mental health diagnoses during the COVID-19 pandemic for older adults in an urban setting.

